# Surfing waves of data in San Diego: sophisticated analyses provide a broad view of human genetic diversity

**DOI:** 10.1186/s13059-014-0562-4

**Published:** 2014-12-17

**Authors:** Mark Reppell, Evan Koch, Benjamin M Peter, John Novembre

**Affiliations:** Department of Human Genetics, University of Chicago, 920 E 58th Street, Chicago, IL 60637 USA; Department of Ecology and Evolution, University of Chicago, 1101 E 57th Street, Chicago, IL 60637 USA

## Abstract

A report on the 64th annual American Society of Human Genetics meeting held in San Diego, USA, 18-22 October, 2014.

Fueled by rapid technological innovations, the ability of geneticists to assay accurately not only genetic data but also gene expression levels, epigenetic markers, biomarker levels and microbiome composition has expanded the field of human genetics vastly. Alongside this expansion, the annual American Society of Human Genetics (ASHG) conference has grown to draw over 6,500 scientific participants with a broad range of interests. Here, we focus on some of the most notable advances made in the area of population genetics that were presented at the 64^th^ annual ASHG meeting. This year, the sophistication of the analysis methods employed was striking, particularly for drawing inferences from subtle differences in human genetic variation.

## Genetic variation data beyond the 1000 Genomes project

For several years, ASHG conferences have been marked by the unveiling of ever-larger and more-complex datasets, and this year was no exception. Goncalo Abecasis (University of Michigan, USA), co-winner of this year’s Curt Stern Award, presented an overview of a landmark achievement in human genetics: the completion of the 1000 Genomes project. The project’s accomplishments in cataloging human variation and pioneering methods for storing, sharing and analyzing massive genetic datasets have been substantial, and the latest phase of analysis presented by Abecasis provided a stunning overview of genetic variation in humans.

Building from Abecasis’ retrospective on the project and its accomplishments, the conference offered an exciting preview of the next stages for human genetics data. For example, the Exome Aggregation Consortium (ExAC) shared how they have jointly analyzed over 90,000 sequenced human exomes and identified hundreds of thousands of segregating loss-of-function variants. As a leader of the ExAC project, Daniel MacArthur (Broad Institute, USA) presented an overview and announced a public website where summary data from 63,000 exomes are being made available. Beyond larger sample sizes, a broader representation of human populations and richer sets of associated phenotype data are also in store for the field. Shop Mallick (Harvard University, USA) presented preliminary findings from the Simons Genome Diversity Project, which has made more than 250 high-coverage human genomes from 129 different populations publicly available. Michael Snyder (Stanford University, USA) gave an update on the Personal ‘Omics’ Profiling project, which is now assaying levels of gene expression, microbiome composition, DNA methylation and multitudinous biomarkers in over 70 individuals, including during experimental perturbations such as controlled weight gain. Another notable development was the emphasis on crowd-sourcing approaches to data collection, and Yaniv Erlich (Whitehead Institute, USA) kicked off a compelling session on the increasing role of these approaches for gathering genetic and phenotypic data in large samples.

## The challenge of large-scale data management

As a natural pairing to discussing new large data-sets, the meeting also highlighted the challenges of handling the ever-larger and more-complex data being generated. Several new tools were introduced, with both academic and commercial computational experts taking the stage. For example, David Glazer (Google, USA) demonstrated the Google Cloud platform as a tool for executing genomic data queries and argued that geneticists should make better use of big-data solutions pioneered in other fields. Two exemplary talks on data management were given by Shane McCarthy (Wellcome Trust Sanger Institute, UK), who addressed issues of file storage and access in the context of the 1000 Genomes data, and Ryan Layer (University of Virginia, USA), who presented the GENOTQ toolkit, designed to balance data compression and computational speed.

## Decoding human population structure

Understanding the history of human populations is a fundamental aspect of the study of human genetics, and this year’s meeting provided exciting new progress on multiple fronts. In particular, a focused session on population history and admixture presented a nice mix of exciting data and news of development of novel methods. Iosif Lazaridis (Harvard University, USA) presented on more than a dozen ancient genomes and how they can be used to infer the complex peopling of the European continent. Stephan Schiffels (Wellcome Trust Sanger Institute, UK), also working with ancient DNA, discussed results from the sequencing of five ancient British samples, remarkably found on the grounds of the Sanger Institute itself. Schiffels’ talk included new methods, based on inter-population rare-variant sharing patterns, that allow inference of very recent timescale events.

Stephen Leslie (Murdoch Childrens Research Institute, Australia) used the hierarchical clustering algorithm fineSTRUCTURE to dissect European population structure at an unprecedented scale, often observing structure where it had not been seen previously and related this structure to historical geo-political boundaries. John Novembre (University of Chicago, USA) presented on a large project to characterize Sardinian population history and used the Sardinian data to demonstrate a novel method of inferring effective migration rates.

Two talks considered population structure in the USA. Yong Wang (Ancestry.com, USA) presented a dataset containing array genotypes for over 500,000 individuals, which he used to show both the differing contributions of ancestral populations throughout the USA and detectable migration patterns between states. Simon Gravel (McGill University, Canada) presented an exploration of population structure within African Americans in the USA and demonstrated how the large-scale northward migrations following emancipation can be traced using identity-by-descent patterns. Overall, the study of population history and structure in humans is showing substantial progress and exciting maturation as methods and data develop.

## Rare variants and purifying selection

A number of talks considered the nature of negative selection on human variation and addressed two important outstanding issues: the recessivity of selective effects in the human genome and the ability to detect rare variant associations when selection and effect sizes are correlated. Daniel Balick (Harvard Medical School, USA) addressed the question of recessivity with a statistic that leverages demographic differences between human populations and showed it successfully predicted recessive selection in genes known to have variants with recessive phenotypic effects. Rare variants are not expected to explain a large proportion of phenotypic variation unless the effect sizes and selection coefficients of variants are correlated. Lawrence Uricchio (University of California San Francisco, USA) used simulations to show that, although this is true, an increased correlation between effect size and selection reduces the power to identify genes in rare-variant association tests.

## Understanding the sex chromosomes

Sex chromosomes, a perennial hot topic in evolutionary genetics that is often overlooked in human genetics, received substantial attention at this year’s meeting both in a focused session and in additional talks. Melissa Wilson Sayers (Arizona State University, USA) presented evidence for the multi-step evolution of mammalian dosage compensation by showing that X-linked genes with recently pseudogenized Y copies are more likely to escape inactivation than genes whose Y copies have been completely lost. She also used population-genetics models and sequence data to show that negative selection in both protein-coding and non-coding regions is necessary to explain the reduced genetic diversity along the Y chromosome. With his talk, Alon Keinan (Cornell University, USA) addressed the role of sex chromosomes in phenotypic variation and disease and introduced XWAS, a toolkit for association studies on the X chromosome.

Two talks presented evidence of faster-X adaptive evolution in apes. Using extensions of the McDonald-Kreitman test, Krishna Veeramah (Stony Brook University, USA) estimated that the rate of adaptive substitution is over fourfold higher along the X chromosome than along autosomes. Mikkel Schierup (Aarhus University, Denmark) demonstrated that, across ape species, ampliconic portions of the X chromosome exhibit regions of greatly reduced diversity. Schierup argued that this reduction was unlikely to be explained by purifying selection and was more consistent with recurrent hard selective sweeps, possibly related to meiotic drive. We expect that future research will extend these findings by distinguishing the roles of dominance, the gene complement of the X chromosome and sexually antagonistic selection in generating patterns of variation on the X chromosome.

## Estimating key rates of change

Crucial to interpreting what genomic structures and patterns of variation tell us about human evolution and disease is understanding the genetic mechanisms of mutation, recombination and gene conversion. Amy Williams (Columbia University, USA) investigated patterns of gene conversion events using genotyping array data from 11 human pedigrees. Williams reported the exciting finding that gene conversion events cluster along haplotypes, a pattern that current models cannot account for. Wendy Wong (Inova Translational Medicine Institute, USA) gave a notable talk on estimating the *de novo* germline mutation rate in humans by using almost 700 deeply sequenced trios. More interesting than the estimate of mean mutation rate itself was the finding of both a significant paternal and maternal age effect. In light of these and other recent findings, Wong is probably correct in asserting that the idea of a per-base/per-generation mutation rate in the absence of accounting for parental age has little meaning beyond a simplifying assumption.

## Concluding remarks

Inevitably, this summary can only scratch the surface of all the compelling work presented at the meeting. In distilling it down, an important takeaway for this set of authors was how, as the field has grown, the ASHG meeting has become increasingly exciting as a venue for the discussion of population-genetic results and methodology. And with analysis methods finally maturing to transform modern genomic-scale data into novel insights, we look forward to further important findings next year in Baltimore.

## Abbreviations

ASHG: American Society of Human Genetics
ExAC: Exome Aggregation Consortium

